# Work e‐mail after hours and off‐job duration and their association with psychological detachment, actigraphic sleep, and saliva cortisol: A 1‐month observational study for information technology employees

**DOI:** 10.1002/1348-9585.12300

**Published:** 2021-11-27

**Authors:** Tomohide Kubo, Shuhei Izawa, Hiroki Ikeda, Masao Tsuchiya, Keiichi Miki, Masaya Takahashi

**Affiliations:** ^1^ National Institute of Occupational Safety and Health Kawasaki Japan; ^2^ Advantage Risk Management Co. Ltd Meguro‐ku Japan

**Keywords:** always‐on work, quick returns, recovery from work, right to disconnect from work, stress hormone

## Abstract

**Objective:**

A sufficient duration of time off after work is necessary to ensure workers’ health. Better quality of off‐job time can also facilitate recovery from fatigue, but its quantitative influence is largely unknown. We aimed to examine how off‐job time quality (as measured by the frequency of emailing after work), and off‐job duration is associated with psychological detachment, actigraphic sleep, and saliva cortisol using a 1‐month observational study.

**Methods:**

The participants were 58 daytime employees working at an information technology company. Sleep actigraphy and saliva cortisol as well as self‐reported outcomes were repeatedly measured for 1 month. Two‐way (work e‐mail frequency × off‐job time) multilevel mixed‐effects linear regression analyses were performed in both continuous and categorical variables.

**Results:**

The frequency of work e‐mailing after hours was significantly associated with self‐reported outcomes and actigraphic sleep quality, while a significant association was not found in cortisol awakening responses and actigraphic sleep duration. A significantly larger cortisol response after awakening was found in shorter, rather than longer, durations of off‐job time. Self‐reported detachment, rumination and carry‐over fatigue showed significant interactions between work e‐mail and off‐job time, suggesting that worse outcomes were found in a higher frequency of work e‐mail even when employees had longer amounts of off‐job time.

**Conclusion:**

Our findings suggest that ensuring the quality and duration of off‐job time is beneficial for recovery from work with sufficient sleep. Specifically, the frequency of e‐mailing after work should be minimized to make recovery complete.

## 1 INTRODUCTION

Ensuring off‐job time is important for recovery from work. According to the effort‐recovery model,^1^ recovery occurs when work demands no longer strain the individual's resources. Mentally detaching from work during off‐job time (i.e., psychological detachment from work) is thought to contribute to recovery.^2^ As information communication technology (ICT) has developed, employees have become mentally bound to work after official hours. Consequently, modern workers are exposed to the potential risks of work anytime, anyplace (the so‐called “always‐on work” approach,^3^ thereby disturbing their opportunity for recovery. Particularly, in terms of psychological detachment, work e‐mailing after hours is anticipated to disturb recovery resources for employees. Earlier studies pointed out that work‐related contact outside of working hours may be connected to health problems.^4^, ^5^, ^6^

Meanwhile, recovery from work is also needed to ensure the daily off‐job rest period. The European Union's (EU) work‐time directive stipulates recovery through “11 consecutive hour daily rest periods between working days”^7^—the so‐called “work‐interval system”. However, evidence regarding the association between off‐job time and health‐related outcomes is lacking for daytime workers. Of the limited data, our previous studies on daytime workers have suggested links between shorter off‐job time and worse health‐related outcomes.^8^, ^9^, ^10^ Hence, ensuring off‐job time (i.e., avoiding overtime work) may be essential to protecting recovery opportunities from work‐induced fatigue among daytime workers.

It should be noted that France introduced the right to disconnect from work in 2017, which forbids employers from taking adverse employment action against workers who do not reply to work‐related texts and e‐mails outside of their normal workday. Given that such a measure is necessary in EU countries, where the work‐interval system has already been introduced, engaging with work e‐mail after hours may increase employees’ *invisible* working hours. The sixth European Working Conditions Survey reported that 22% of employees work in their free time to meet work demands at least several times per month.^11^ Recently, EU has been discussing whether the right to disconnect from work should be a fundamental right across the 27 member states.^12^

At the moment, some findings suggest a cross‐sectional association between business e‐mailing after working hours and health‐related outcomes. Nevertheless, objective data are still lacking because the assessments in previous research were often made with self‐reported measurements.^4^, ^5^, ^6^, ^13^, ^14^ Moreover, the interaction between work e‐mail frequency and off‐job duration is also unclear. In other words, some employees work in their off‐job time by means of ICT with sufficient off‐job time, and others work outside their official hours without sufficient off‐job time. Considering the intensity of work, it is likely that the latter case would be more problematic. Our hypothesis here is that less frequent work e‐mailing after hours coupled with longer off‐job time could be linked to employees’ better psychological detachment, thereby ameliorating the recovery process. At the same time, it is expected that, even when employees have sufficient off‐job time, higher frequency of work e‐mailing after hours could deteriorate their health‐related outcomes. However, available data are limited to knowledge about the workload difference. To fill the gap, there is a need for a full understanding of the physiological, behavioral, and psychological health impacts caused by job‐related e‐mailing after working hours and during off‐job time. Thus, we aim to examine how the frequency of work e‐mailing after hours and off‐job time are associated with psychological outcomes, sleep actigraphy and saliva cortisol as a stress hormone in a 1‐month observational study.

## 2 METHODS

### 2.1 Participants

Since our interest was the association between the use of ICT and work, we looked for information technology companies to collaborate with us by using the community of the Japan Society for Occupational Health. As a result, we found a collaborating company that was located in Tokyo and had more than 2000 employees. Then, we recruited possible participants through the counter partner who worked on the company as an occupational health nurse. We set the selection criteria as (1) employees ranging in age from 20 to 50 years, (2) employees who could participate for 1 month. In addition, the incentive for participation was only to provide feedback through which they could know the status of their health. Thus, we did not provide any monetary reward for participation in this study. A total of 68 employees were selected as participants. Of them, 10 employees did not record the primary data parameter (i.e., frequency of work e‐mail after hours) at all. Therefore, the data of 58 participants (31 males/27 females, mean = 39.3 ± 6.2 years) were analyzed in this study. Their other characteristics were shown in Table 1. The local institutional review board reviewed and approved the study protocol (H26‐1‐02). All participants provided written informed consent.

**TABLE 1:** Demographic data of the participants

*N*	58
Age (year, mean ± SD)	39.3 (6.2)
Gender (% female)	46.6
Marriage (%)	63.8
One‐way commuting time (min, median [IQR])	45.0 (35.0–60.0)
Self‐reported daily working hours (h, median [IQR])	9.5 (9.0–11.0)
Medication treatment (%)^a^	44.8
Work position (% manager)	12.1
	Mean ± SD
Self‐reported parameters	
Carry‐over fatigue (mm)	59.7 (25.0)
Detachment (mm)	40.5 (28.3)
Rumination (mm)	49.7 (29.5)
Sleep parameters	
TST (h)	6.1 (1.8)
WASO (min)	18.2 (27.0)
SE (%)	95.5 (5.8)
Physiological parameter	
Δ Cortisol (nmol/L)	12.7 (22.1)

Abbreviations: SE, sleep efficiency; TST, total sleep time; WASO, wake time after sleep onset.

^a^ The data include taking medicine or being sick (e.g., cold, etc.), which could affect the results of cortisol response.

### 2.2 Study design

**FIGURE 1:**
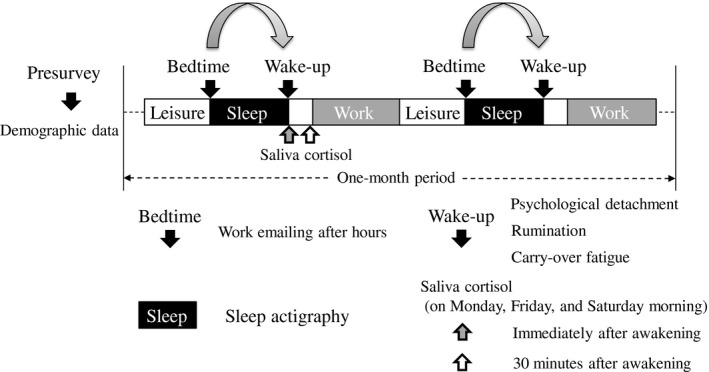
Study design

We conducted a 1‐month observational study to examine the daily associations between the frequency of job‐related e‐mailing after work and health outcomes from October to December 2015. During the study period, the participants were required to wear sleep actigraphs (Zzz‐Logger; Ambulatory Monitoring Inc.) during sleep periods and to use a tablet fatigue app to measure e‐mail frequency, off‐job time, and self‐reported outcomes by themselves (Figure 1). The fatigue app that we have developed has some functions to easily measure fatigue‐related parameters, such as self‐reported outcomes, a performance test, and a life log.^13^ The app is provided on our institutional web‐site (https://www.jniosh.johas.go.jp/publication/application/application_2020_01.html). In addition, saliva samples were collected upon awakening for the objective assessment of stress.

### 2.3 Demographic data

Participants were asked about their demographic data before the beginning of this study with a pre‐survey questionnaire, including their age, gender, commuting time, marital status, and medical treatments.

### 2.4 Work e‐mailing after hours

We sought to measure the frequency of work‐related e‐mailing after work using any type of information and technology devices, including personal computers, smartphones, tablets, and others. However, the appropriate referent scale was unavailable, so we decided to adopt the visual analogue scale (VAS) in this study. Then, the participants were required to answer the question, “How often do you contact your coworkers (or clients) with job‐related e‐mail outside of working hours?” (0 = “*not at all*”, 100 = “*many*”). Also, the measurement was conducted at bedtime using the fatigue app. The data regarding the VAS‐measured work e‐mailing frequency were divided into two levels (e‐mail frequency [high, low]) according to the median (median e‐mail frequency = 38 mm), when analyzing the data categorically.

### 2.5 Off‐job time

Based on the daily log measured by the fatigue app, off‐job times were calculated as the interval from the end of working hours to the start of working hours (including commute time), and analyzed using not only weekday but also weekend. In Japan, official working hours including rest period is set as 9 h per day. Hence, short off‐job time (as overtime work) was defined as <15 h off‐job time, while long off‐job time (as non‐overtime work) was defined as 15 h off‐job time or more. Then, we divided these data into two levels (off‐job time [short = less than 15 h, long = 15 h or more]) based on whether employees worked overtime. Also, the data were analyzed as a categorical variable.

### 2.6 Self‐reported outcomes

The participants were required to conduct VAS measurements regarding psychological detachment from work (“I do not think about work at all,” 0 = *Definitely no*, 100 = *Definitely yes*), carry‐over fatigue (“I carry over work‐induced fatigue,” 0 = *not fatigued at all*, 100 = *extremely fatigued*), and rumination (“Since I repeatedly think about work, I cannot get it out of my head,” 0 = *Definitely no*, 100 = *Definitely yes*) upon awakening by using a fatigue app. VAS‐measured psychological detachment was highly correlated with the recovery experience questionnaire^15^ which assesses how individuals’ recover from work during leisure time (i.e., psychological detachment, relaxation, mastery and control) at the end of this study (*r* = .675, *P* < .001). Therefore, VAS‐measured psychological detachment could be a reasonable proxy for the established measure.

### 2.7 Sleep actigraphy

Sleep was measured by means of an actigraph, which was secured to the participants’ non‐dominant wrist during the period of study. The epoch length was set at 1 min. The total sleep time (TST) and sleep efficiency (SE; the percent of time scored as sleep during the sleep period) as well as the wake time after sleep onset (WASO), were calculated to examine the quantity and quality of sleep, using AW2 ver.2.6 (Ambulatory Monitoring Inc.).

### 2.8 Saliva cortisol

Saliva samples were collected upon awakening and 30 min after awakening with a Salivette (Sarstedt Ltd) polypropylene and polyethylene polymer swab, based on the previous study method.^16^ The participants were required to place the swab under their tongue for at least three minutes to obtain the sample. They were also instructed to refrain from eating, drinking, or brushing their teeth for 30 min after awakening. Saliva collections were conducted three times per week (on Monday, Friday, and Saturday morning). According to earlier findings,^17^ the cortisol awakening response is a useful indicator of hypothalamic–pituitary–adrenal activity. Therefore, the delta value between the samples taken immediately after awakening (T1) and 30 min after awakening (T2) (i.e., T2−T1) was analyzed to examine the cortisol awakening response. The concentration of cortisol in the saliva was determined by an enzyme immunoassay using an ELISA Kit (IBL International). The inter‐ and intra‐assay variations were below 7.3% and 9.3%, respectively. Before the beginning of this study, we instructed the participants to ensure that they clearly understood the sampling protocol, and we addressed any of their doubts. Written instructions for the saliva collection protocol were handed out. Also, the specialist (SI) conducted the data analyses in our institute's laboratory.

### 2.9 Data analyses

Repeated daily measurements (i.e., work e‐mailing after work, off‐job time, self‐reported outcomes, sleep actigraphy, and cortisol awakening response) were nested within individuals. Because these data were based on a 1‐month observational study design in which an individual's responses over time are correlated with each other, we adopted a multilevel approach. Covariates were modelled at the in‐between level.

Data on postwork e‐mail frequency and off‐job time were divided into two levels (e‐mail frequency [high, low], off‐job time [short, long]). Multilevel mixed‐effects models were used to evaluate the effects of postwork e‐mail frequency and off‐job time on fatigue‐related outcomes. Then, postwork e‐mail frequency and off‐job time were included as a fixed factor, and each participant was entered as a random factor. Based on the STROBE statements, especially number 11,^18^ the fixed factors of off‐job time and postwork e‐mail frequency were analyzed by setting both continuous and categorical variables. Age, gender, weekday day (from Monday to Sunday), marital status, and commuting time might have affected the main outcomes^19^; therefore, these variables were treated as covariates. In addition, medical treatment was included as the covariate in the analysis of the cortisol awakening response. Also, our research interest is to know about the difference in work intensity due to job‐related e‐mailing after work under the same length of off‐job time. Thus, planned comparisons with the Bonferroni method were performed to examine whether there was a significant difference in sleep actigraphy, self‐reported data, and saliva cortisol among four conditions when analyzing the fixed factor as a categorical variable: (A) shorter off‐job time with lower e‐mail frequency; (B) shorter off‐job time with higher e‐mail frequency; (C) longer off‐job time with lower e‐mail frequency; and (D) longer off‐job time with higher e‐mail frequency. Statistical analyses were performed using Stata/CI 14.0 for Windows, and the statistically significant difference was set at *P* < .05.

## 3 RESULTS

Table 2 summarizes the result of our multilevel analyses regarding self‐reported outcomes, sleep parameters, and cortisol awakening response when analyzing the effect of e‐mail frequency and off‐job time with both continuous and categorical variables.

**TABLE 2:** Results from linear mixed‐effects models predicting parameters

E‐mail frequency	Continuous variable				Categorical variable			
	mm				Low (ref.) vs. High			
Self‐reported parameters	Beta	95% CI		*P*	Beta	95% CI		*P*
Carry‐over fatigue (mm)	−0.034	−0.134	0.066	.501	−2.729	−7.769	2.312	.289
Detachment (mm)	**−0.126**	**−0.232**	**−0.020**	.**020**	−1.434	−6.729	3.860	.595
Rumination (mm)	**0.143**	**0.045**	**0.242**	.**004**	3.168	−1.728	8.065	.205
Sleep parameters								
TST (h)	−0.005	−0.011	0.002	.166	0.164	−0.172	0.500	.338
WASO (min)	0.029	−0.074	0.132	.581	3.675	−1.519	8.869	.165
SE (%)	−0.005	−0.026	0.016	.626	−0.709	−1.774	0.356	.192
Physiological parameter								
Δ Cortisol (nmol/L)	−0.075	−0.262	0.112	.429	−5.228	−14.541	4.085	.271

Off‐job time	Continuous variable				Categorical variable			
	h				<15 h (ref.) vs. ≥15 h			
Self‐reported parameters	Beta	95% CI		*P*	Beta	95% CI		*P*
Carry‐over fatigue (mm)	**−0.404**	**−0.484**	**−0.325**	**<.001**	**−23.380**	**−28.001**	**−18.760**	**<.001**
Detachment (mm)	**0.120**	**0.034**	**0.206**	.**006**	**9.442**	**4.565**	**14.319**	**<.001**
Rumination (mm)	**−0.110**	**−0.190**	**−0.031**	.**006**	**−10.503**	**−15.005**	**−6.002**	**<.001**
Sleep parameters								
TST (h)	**0.007**	**0.001**	**0.012**	.**013**	**0.783**	**0.472**	**1.094**	**<.001**
WASO (min)	0.041	−0.042	0.125	.329	2.097	−2.696	6.889	.391
SE (%)	−0.003	−0.020	0.014	.693	−0.017	−0.993	0.958	.972
Physiological parameter								
Δ Cortisol (nmol/L)	−0.107	−0.280	0.066	.225	**−10.057**	**−19.413**	**−0.701**	.**035**

Interaction (e‐mail frequency × off‐job time)	Continuous variable				Categorical variable			
Self‐reported parameters	Beta	95% CI		*P*	Beta	95% CI		*P*
Carry‐over fatigue (mm)	**0.004**	**0.002**	**0.006**	**<.001**	**14.866**	**8.649**	**21.083**	**<.001**
Detachment (mm)	−0.001	−0.003	0.001	.312	**−10.367**	**−16.959**	**−3.774**	.**002**
Rumination (mm)	0.001	−0.001	0.003	.272	**9.451**	**3.366**	**15.536**	.**002**
Sleep parameters								
TST (h)	0.001	−0.001	0.001	.061	−0.117	−0.550	0.316	.596
WASO (min)	0.001	−0.001	0.003	.451	2.689	−3.983	9.360	.430
SE (%)	−0.001	−0.001	0.001	.689	−0.329	−1.687	1.029	.635
Physiological parameter								
Δ Cortisol (nmol/L)	0.001	−0.003	0.004	.688	5.851	−5.890	17.592	.329

Note

Off‐job time and e‐mail frequency were included as a fixed factor, while the participant was entered as a random factor, and covariates of age, gender, marriage, day (from Monday to Sunday), marital status, and commuting time were included. In addition, medical treatment was included as the covariates in the analysis of the cortisol awakening response. β represents the regression coefficient for the fixed effect model, and the coefficients in bold show the significant level of difference. Values in bold indicate significant differences.

Abbreviations: 95% CI, 95% confidence interval; SE, sleep efficiency; TST, total sleep time; WASO, wake time after sleep onset.

### 3.1 Self‐reported variables

**FIGURE 2:**
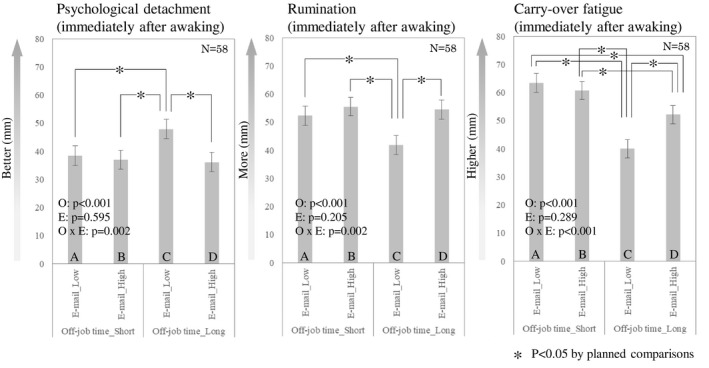
Self‐reported parameters associated with the frequency of work emailing after hours and the amount of off‐job time

Figure 2 represents the effects of work e‐mailing after hours and off‐job time on self‐reported outcomes in terms of the categorical variables. In a continuous variable, VAS‐measured psychological detachment and rumination showed a significant main effect on the e‐mail frequency (beta = −0.126 [95% CI; −0.232, −0.020], *P* = .020, beta = 0.143 [95% CI; 0.045, 0.242], *P* = .004, respectively) (Table 2). In other words, psychological detachment decreased by about 0.12 mm for each 1 mm of e‐mail frequency, while rumination increased by about 0.14 for each 1 mm of e‐mail frequency. On the other hand, no significant findings were found in the categorical variable. Regarding off‐job time, there were significant differences in all self‐reported outcomes for both continuous and categorical variables. In a continuous variable, carry‐over fatigue and rumination significantly decreased by about 0.40 and 0.11 mm for each hour of off‐job time (beta = −0.404 [95% CI; −0.484, −0.325], *P* < .001, beta = −0.110 [95% CI; −0.190, −0.031], *P* = .006, respectively), while psychological detachment significantly increased by about 0.12 mm for each hour of off‐job time (beta = 0.120 [95% CI; 0.034, 0.206], *P* = .006). Meanwhile, compared with <15 h of off‐job time (reference), carry‐over fatigue and rumination significantly decreased in 15 h off‐job time or more (beta = −23.38 [95% CI; −28.001, −18.760], *P* < .001, beta = −10.503 [95% CI; −15.005, −6.002], *P* < .001, respectively), while psychological detachment significantly increased in the same condition (beta = 9.442 [95% CI; 4.565, 14.319], *P* < .001). Regarding the interaction between e‐mail frequency and off‐job time, carry‐over fatigue showed significant difference in the continuous variable (beta = 0.004 [95% CI; 0.002, 0.006], *P* < .001). On the other hand, significant differences were found in carry‐over fatigue (beta = 14.866 [95% CI; 8.649, 21.083], *P* < .001), psychological detachment (beta = −10.367 [95% CI; −16.959, −3.774], *P* = .002), and rumination (beta = 9.451 [95% CI; 3.366, 15.536], *P* = .002) in the categorical variable. As shown in Figure 2, planned comparison indicated that significant differences were observed in psychological detachment and rumination between C and A, B, D (*P* < .05). Carry‐over fatigue significantly differed between C and A, B, D, as well as between A and D (*P* < .05, respectively). Notably, those results indicated that a significantly better psychological detachment, lower rumination, and lower carry‐over fatigue were observed in lower e‐mail frequency with longer off‐job time (i.e., C) compared with other conditions.

### 3.2 Actigraphically measured sleep

**FIGURE 3:**
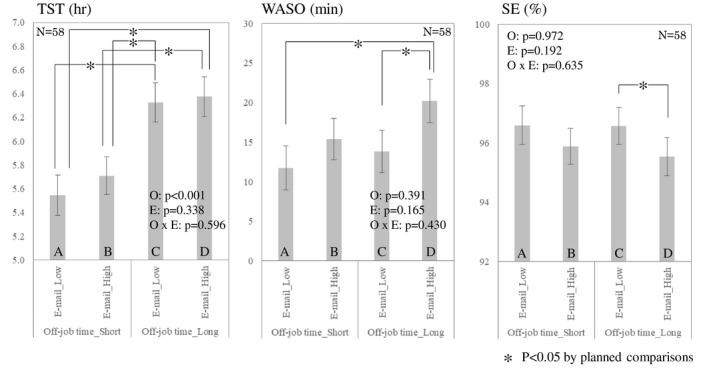
Actigraphiycally measured sleep parameters associated with the frequency of work emailing after hours and the amount of off‐job time

Figure 3 shows the effects of work e‐mailing after hours and off‐job time on sleep parameters in terms of categorical variables. Regarding e‐mail frequency, three sleep parameters (TST, SE and WASO) showed no significant differences in both variables. On the other hand, a significant main effect of off‐job time was found in both variables (continuous variable: beta = 0.007 [95% CI; 0.001, 0.012], *P* = .013; categorical variable: beta = 0.783 [95% CI; 0.472, 1.094], *P* < .001). Namely, TST increased by about 30 s for each hour of off‐job time in the continuous variable, while about 47 min of TST increased by 15 h or more compared to <15 h (reference) in the categorical variable. There was no significant interaction between e‐mail frequency and off‐job time. As shown in Figure 3, planned comparison indicated that significant differences were observed in TST between A and C, D, as well as between B and C, D (*P* < .05, respectively). Regarding WASO, significant differences between A and D (*P* < .05) and C and D (*P* < .05) were found. In addition, SE showed significant differences between C and D (*P* < .05). In particular, these results suggested that a significantly shorter WASO and higher SE were found in longer off‐job time with lower e‐mail frequency (i.e., C) compared with other conditions.

### 3.3 Saliva cortisol awakening response

**FIGURE 4:**
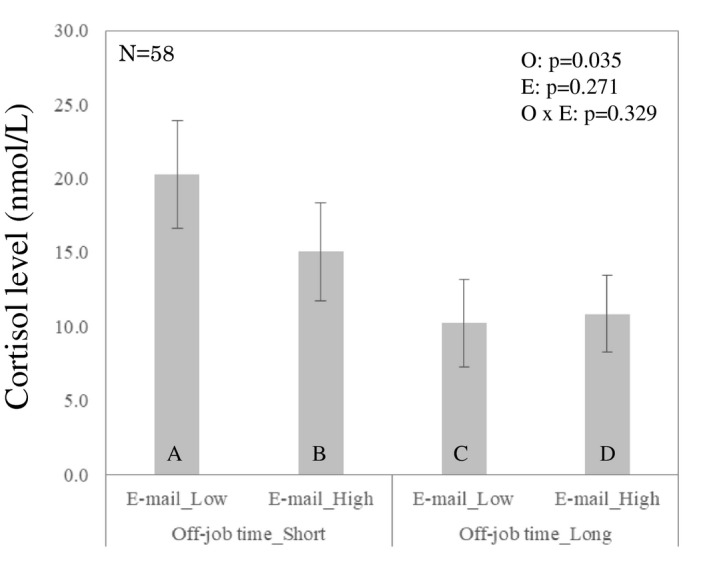
Saliva cortisol awakening response (the delta value between the samples taken immediately after awakening and 30 min after awakening) associated with the frequency of work emailing after hours and the amount of off‐job time

Table 2 and Figure 4 indicate that no significant difference regarding e‐mail frequency was found in either the continuous or categorical variables. Meanwhile, a significant main effect of off‐job time was observed in the categorical variable (beta = −10.057 [95% CI; −19.413, −0.701], *P* = .035), suggesting that the cortisol awakening response significantly decreased by about 10.06 nmol/L for each hour of off‐job time. The interaction between e‐mail frequency and off‐job time did not show a significant difference in either the continuous or categorical variables. Moreover, planned comparison did not show any significant difference (Figure 4).

## 4 DISCUSSION

This study aimed to examine how work e‐mailing after hours and off‐job time influence health‐related outcomes within a 1‐month observational study design. Our results showed that better outcomes were observed with lower frequency of work e‐mailing after hours and longer off‐job time. Also, deteriorated self‐reported outcomes and sleep quality were found in higher frequency e‐mailing even though off‐job time was a longer condition. Moreover, a significantly higher quality of sleep (i.e., WASO and SE) was found in lower work e‐mail frequency than in higher work e‐mailing frequency under the condition of longer off‐job time (Figure 3). Also, a significantly lower amount of cortisol was found in relation to longer, rather than shorter, off‐job time (beta = −10.057 nmol/L), although no significant difference was obtained in e‐mail frequency.

It should be noted that shorter off‐job times led to significantly higher levels of cortisol response, shorter sleep duration, worse self‐reported outcomes. This finding is in line with the previous findings suggesting that insufficient off‐job time could lead to deteriorated health and safety outcomes.^20^, ^21^, ^22^, ^23^ Furthermore, the findings support our hypothesis regarding the link between shorter off‐job time and deteriorated sleep. However, as far as we know, the present findings may be the first to show the relationship between longer off‐job time and less physiologically‐measured stress (i.e., saliva cortisol awakening response), highlighting the importance of preventing overtime work among employees. Notably, the Japanese government has set a goal of getting at least 10% of companies to launch the “work‐interval system” to avoid quick returns (i.e., 11 h or less between two consecutive shifts).^24^ Therefore, our findings provide partial empirical support for the work‐interval system, although this study did not directly examine the association between quick returns and health‐related outcomes.

In the present study, job‐related e‐mailing after working hours negatively affected the quality of sleep, rumination, carry‐over fatigue and psychological detachment (Figures 2 and 3, Table 2). These findings are consistent with our hypothesis and a previous study examining the links between work‐related smartphone use and psychological detachment.^14^ If work e‐mailing after hours was linked to apprehension regarding work, plausibility of our findings would be supported by previous experiments, showing that high apprehension regarding the subsequent workday was associated with less slow‐wave sleep, more stage 2 sleep, difficulties waking, and poor self‐reported sleep quality.^25^ It was also reported that a high level of work‐related rumination could induce sleep disturbances among workers.^26^ As we expected, it is probable that employees who often e‐mail after working hours frequently think about work and have difficulty psychologically detaching from work. Deteriorating quality during subsequent sleep may disturb the recovery process. This possible pathway is supported by earlier research.^27^

Great care is needed, however, when interpreting the results of sleep efficiency and WASO in this study. A decrease of approximately 1% in sleep efficiency and a 5 min increase in WASO occurred with a high frequency of work e‐mailing after hours compared to the low‐frequency condition (see Figure 3). These differences should be seen as minor, given actigraphic data for insomnia patients (e.g., 86.4 ± 9.3% in sleep efficiency and 45.2 ± 38.2 min in WASO^28^). However, in this study, the amount of lower sleep efficiency and longer WASO than in the insomnia patient data was higher in the high‐frequency condition (sleep efficiency = 8.3%, WASO = 12.0%) than in the low‐frequency condition (sleep efficiency = 2.9%, WASO = 5.3%). Hence, frequently e‐mailing after work may be a potential risk factor for developing future health problems. On the other hand, a 7‐day actigraphy study revealed that daily stress from friends or family resulted in an approximate 4% decrease in sleep efficiency.^29^ Given those findings, the magnitude of sleep parameters in this study could not be negligible although the participants were healthy workers.

Moreover, it is important to address the question of why the negative impacts of work e‐mailing after hours were not observed in the cortisol awakening response. We may propose two possibilities to answer the above question. First, the content of work e‐mailing may be a confounding factor. The e‐mails in this study may mix contents, resulting in the emergence of positive or negative feelings. Second, previous research examined the association between work‐related smartphone use and work–family conflict in terms of a work–home segmentation preference.^13^ In the study, an individual who preferred to separate work and private life was defined as a segmentor, and one who preferred to integrate work and private life was defined as an integrator. Integrators experienced more work–family conflict on days when they used their smartphone less intensively for work‐related activity during off‐job times. To sum up, this finding suggests that the impact of work‐related smartphone use after hours may depend on individual preference.

Finally, we have to discuss about the relative strength between e‐mail frequency after work and off‐job time for predicting outcomes. As shown in Table 2, five outcomes—TST, carry‐over fatigue, detachment, rumination, and cortisol—showed significant main effects of off‐job time, while two outcomes—detachment and rumination—were significant in e‐mail frequency after work. Off‐job time was significantly linked to physical, behavioral and psychological outcomes, though only psychological outcomes were significant for e‐mail frequency after work. These results, therefore, suggest that the relative magnitude could be higher in off‐job time than in e‐mail frequency after work. These findings are plausible because the length of off‐job time is directly related to physical time that allows employees to sleep at home. During the period of off‐job time, employees spend more than half of the total time to sleep at home. On the other hand, the physical time that employees spend e‐mailing after work will be much shorter compared with sleep duration. However, given that only one e‐mail may trigger insomnia, further investigations are needed to determine whether work e‐mailing after hours could affect future mental health among employees.

### 4.1 Limitations of this study

Several limitations should be addressed here. First, the measurement of e‐mail frequency in this study was based on self‐evaluation with the VAS method because the appropriate scale was unavailable. In addition, we guess that the workload of e‐mailing after work may be more closely related to individual perception than to actual e‐mail conditions (e.g., number and timing). Hence, the current method based on self‐evaluation may be effective for measuring the workload from e‐mailing after work, although it is important to examine actual e‐mail conditions in future research. Furthermore, this study used VAS method to measure self‐reported outcomes because of the feasibility for repeated sampling during 1‐month period. Then, the question here to be addressed is whether VAS method (esp., psychological detachment) could be valid. However, given that significant correlation between VAS‐measured psychological detachment and established questionnaire^15^ at the end of this study was observed (*r* = .675, *P* < .001), the usefulness of VAS‐measured psychological detachment could be secured. Second, this observational study did not have a control or other occupation group that may have been useful for comparison. Therefore, further research with control group, such as an intervention study design, should be conducted in next step. Third, the participants were required to measure their saliva by themselves without our observation. Given the guideline for assessing cortisol awakening response,^30^ the effect of a delayed initial sample after awakening likely affected our data. However, our participants were instructed to conduct VAS measurements immediately after awakening and to record their measurement times in the fatigue app. They were also required to record their saliva measurement times. Then, we excluded the data if big differences were apparent between fatigue‐app timing and self‐reported timing of saliva collection. The bias resulting from such a delayed initial sample could thus be partially controlled by the procedure. Forth, of the 68 participants, 10 dropped out in this study. One possible reason may be that the participation incentive was only a feedback report providing them with their health status but no monetary incentive. Therefore, there may be a selection bias in that employees with high motivation participated in this study. Finally, the generalizability of these findings should be examined in further research as the targeted population consisted of participants from a single occupation—information technology workers.

## 5 CONCLUSION

The findings reported here provide new insights into how work e‐mailing after hours, and off‐job time are associated with stress and sleep among workers. As the negative relationship between shorter off‐job time and higher physiologically measured stress levels was observed in this study, our findings highlight the importance of a work‐interval system to counter overworking. Additionally, given that the negative influences of higher frequent job‐related e‐mailing after work were found even when off‐job time was longer, our findings suggest that employees who often e‐mail outside of working hours could repeatedly think about work, thereby disturbing their opportunity to recover from work. Therefore, the study reveals the benefits of implementing the right to disconnect from the workplace to protect workers’ recovery opportunities. Specifically, the important message from this study is that the frequency of e‐mailing after work should be minimized to ensure complete sleep recovery in the digitalized working world. Moreover, the spread of remote work in relation to the COVID‐19 pandemic has blurred the boundaries between work and private life. Given this situation, protective countermeasures against “always‐on work” may be more necessary in new normal life than before the COVID‐19 pandemic.

## DISCLOSURE

*Approval of the research protocol*: This study was supported by the National Institute of Occupational Safety and Health, Japan (N‐P26‐01). *Informed Consent*: All participants provided written informed consent. *Registry and the registration no*. *of the study*/*trial*: N/A. *Animal studies*: N/A. *Conflict of interest*: We declare no conflicts of interest, including the fatigue app or device with which we developed and reported data.

## AUTHOR CONTRIBUTIONS

All authors contributed to planning the design of this study. TK, SI, HI, and MT explained this the design of this study to possible participants and collected the workplace data. TK, SI, and HI analyzed the data. TK prepared the first version of the manuscript. All authors critically revised the manuscript and approved the final version.

## Data Availability

The data are not available because we did not inform the participants of data transparency, and we declare the possibility to the institutional review board.
